# *APOE *polymorphism is associated with lipid profile, but not with arterial stiffness in the general population

**DOI:** 10.1186/1476-511X-9-128

**Published:** 2010-11-08

**Authors:** Rafael O Alvim, Silvia RS Freitas, Noely E Ferreira, Paulo CJL Santos, Roberto S Cunha, José G Mill, José E Krieger, Alexandre C Pereira

**Affiliations:** 1 Heart Institute (InCor), University of São Paulo Medical School, Brazil; 2 Department of Physiology, Espírito Santo Federal University, Brazil

## Abstract

**Background:**

Cardiovascular diseases (CVD) are the main cause of death and disability in developed countries. In most cases, the progress of CVD is influenced by environmental factors and multifactorial inheritance. The purpose of this study was to investigate the association between *APOE *genotypes, cardiovascular risk factors, and a non-invasive measure of arterial stiffness in the Brazilian population.

**Methods:**

A total of 1493 urban Brazilian individuals were randomly selected from the general population of the Vitoria City Metropolitan area. Genetic analysis of the *APOE *polymorphism was conducted by PCR-RFLP and pulse wave velocity analyzed with a noninvasive automatic device.

**Results:**

Age, gender, body mass index, triglycerides, creatinine, uric acid, blood glucose, blood pressure phenotypes were no different between ε2, ε3 and ε4 alleles. The ε4 allele was associated with higher total-cholesterol (p < 0.001), LDL-C (p < 0.001), total-cholesterol/HDL-C ratio (p < 0.001), LDL/HDL-C ratio (p < 0.001), lower HDL-C values (p < 0.001) and higher risk to obesity (OR = 1.358, 95% CI = 1.019-1.811) and hyperuricemia (OR = 1.748, 95% CI = 1.170-2.611). Nevertheless, pulse wave velocity (p = 0.66) measures were no different between genotypes. The significant association between APOE genotypes and lipid levels persisted after a 5-year follow-up interval, but no interaction between time and genotype was observed for lipids longitudinal behavior.

**Conclusion:**

The ε4 allele of the *APOE *gene is associated with a worse lipid profile in the Brazilian urban population. In our relatively young sample, the observed effect of *APOE *genotype on lipid levels was not translated into significant effects in arterial wall stiffness.

## Background

Cardiovascular diseases (CVD) are the main cause of death and disability in developed countries. In most cases, the progress of CVD is influenced by multifactorial inheritance and environmental factors [1-4]. Lipoprotein disorders such as elevated low-density lipoprotein cholesterol (LDL-C ≥ 160 mg/dL), low high-density lipoprotein cholesterol (HDL-C < 40 mg/dL) and elevated concentration of triglycerides (TG ≥ 150 mg/dL) are considered significant risk factors in the pathogenesis of CVD [5].

Apolipoprotein E (ApoE) is a glycoprotein that plays a fundamental role in the lipid metabolism. ApoE participates in the clearance of chylomicron remmants and very low-density lipoprotein (VLDL) by serving as a ligand for LDL receptors[6]. It is also important for intestinal cholesterol absorption[7] and plasma lipid maintenance[8]. The *APOE *gene, located on chromosome 19[9], is composed by three alleles (ε2, ε3 and ε4) that give rise to six different genotypes (ε2/2, ε2/3, ε2/4, ε3/3, ε3/4, and ε4/4)[6]. The ε3 allele differs from the ε2 allele by an amino acid substitution of arginine for cysteine at codon 158, while the ε4 differs from ε3 by a substitution of arginine for cysteine at residue 112[10-12]. Many studies assessing the role of *APOE *polymorphism on plasma lipids have shown that the presence of the ε4 allele is associated with elevations in LDL-C, while the presence of ε2 is associated with decreased levels of LDL-C[13]. Moreover, some studies have reported that the ε4 allele is associated with coronary heart disease[14] although most of these have been carried out in male subjects.

After the discovery of the *APOE *gene and knowledge of its genetic variants, several studies have demonstrated the association between the *APOE *polymorphisms and chronic conditions, such as Alzheimer's disease[15], age-related cognitive decline[16], osteoporosis[17], breast cancer[18], end-stage renal disease[19], atherosclerosis[8], diabetes[20], coronary disease[21] and longevity[22]. Based on these, we aimed to assess the relation between *APOE *genotype groups with the prevalence of the major CVD risk factors and its possible association with the evolution of the studied phenotypes in a longitudinal study of Brazilian subjects randomly selected from an ethnically mixed urban population.

Our hypothesis was that individuals carrying the ε4 allele had a worse lipid profile when compared with ε2 and ε3 alleles carriers of the *APOE *polymorphism and that this worse profile would be translated into significantly different measures of arterial stiffness.

## Methods

### Study Design and Participants

A cross-sectional study of risk factors for cardiovascular diseases was performed in the urban population of the Vitória city, Brazil, using the WHO-MONICA project guidelines[23]. In the first stage of the study, conducted in 1999, 1493 Brazilians of either gender, aged 25 to 64 years were chosen according to the nearest birthday[24]. All participants were submitted to complete clinical and laboratorial investigations for CVD risk factors.

This study was approved by the Ethics Committee for Research on Human Subject of the Espírito Santo Federal University, and all subjects gave written informed consent to participate.

### Risk Factors Assessment

#### Anthropometrical Investigations

Weight and height were measured according to a standard protocol, with participants wearing light clothing and no shoes. Height was measured in centimeters and weight in kilograms using a calibrated balance. Body mass index (BMI) was calculated and obesity defined as BMI ≥ 30Kg/m^2^[25]. Individuals who had ever smoked more than five cigarettes per day for the last year were classified as smokers[26]. Participants were also submitted to an ethnic classification according to a validated questionnaire for the Brazilian population[27]. Subjects were classified as Caucasian or Afro-descendent according to a set of phenotypic characteristics (skin color, hair texture, shape of the nose and aspect of the lip). On the basis of these characteristics, mulattos are considered racially mixed subjects.

#### Blood Pressure Phenotype Determination

Blood pressure was measured in the sitting position with the use of a standard mercury sphygmomanometer on the left arm after 5 minutes' rest. The first and fifth phases of Korotkoff sounds were used for systolic (SBP) and diastolic pressure (DBP), respectively. The SBP and DBP were calculated from two readings with a minimal interval of 10 minutes. Hypertension was defined as mean SBP ≥ 140 mmHg and/or DBP ≥ 90 mmHg or use of anti-hypertension drugs [28]. Pulse pressure (PP) was the difference between SBP and DBP. The mean blood pressure (MBP) was calculated as the mean pulse pressure added to one-third of the DBP.

#### Pulse wave measurements

The Carotid-femoral pulse wave velocity (PWV) was analyzed with a noninvasive automatic device (Complior; Colson; Garges les Gonesses, France) by an experienced observer blinded to the clinical characteristics. Briefly, common carotid artery and femoral artery pressure waveforms were recorded noninvasively using a pressure- sensitive transducer (TY-306-Fukuda; Fukuda; Tokyo, Japan). The distance between the recording sites (D) was measured, and PWV was automatically calculated as PWV = D/t, where (t) means pulse transit time. Thirty measurements were repeated over 10 different cardiac cycles, and the mean is used for the final analysis. Because systolic BP has direct influence on PWV, we also adjusted PWV for the mean systolic BP in all groups. The validation of this automatic method and its reproducibility has been previously described[29].

#### Biochemical Measurement

Blood glucose, TG, lipoprotein fractions and uric acid were assayed by standard techniques in 12-h fasting blood samples [30].This study, diabetes was defined as fasting glucose ≥ 126 mg/dL or use of hypoglycemic drugs. Abnormal biochemical levels were identified when total-cholesterol (TC) ≥ 200 mg/dL, TG ≥ 150 mg/dL, LDL-C ≥ 160 mg/dL, HDL-C < 40 mg/dL and uric acid ≥ 7.0 mg/dL[5,31].

### DNA Extraction and *APOE *genotyping

**Figure 1:**
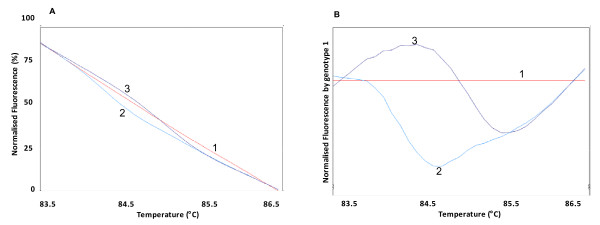
**Genotyping of rs7412 through HRM analysis**. A: Normalized fluorescence by temperature. B: Normalized fluorescence (based on genotype 1) by temperature. 1: wild-type genotype; 2: heterozygous genotype; 3: homozygous genotype for the APOE rs7412 polymorphism.

Genomic DNA was extracted from leukocytes in samples of whole blood, following a standard salting-out technique[32]. Genotypes were detected by polymerase chain reaction followed by restriction fragment length polymorphism analysis as previously described[33]. In addition, we have genotyped samples for the APOE polymorphisms (rs7412 and rs429358) by an additional method (HRM analysis - high resolution melting). Briefly, PCR with a fluorescent DNA-intercalating SYTO9^® ^(Invitrogen, Carlsbad, USA) was performed using the primer sequences 5'-GCCGATGACCTGCAGAAG-3' and 5'-CACGCGGCCCTGTTCCAC-3' (fragment size 117 pairs base) and 5'-GCGGACATGGAGGACGTG-3' and 5'- AGCTCCTCGGTGCTCTGG-3' (fragment size 83 pairs base), for rs7412 and rs429358, respectively. In the HRM phase, Rotor Gene 6000^® ^(Qiagen, Courtaboeuf, France) measured the fluorescence in each 0.1°C temperature increase in the range of 70-94°C. Melting curves were generated by the decrease in fluorescence with the increase in the temperature; nucleotide changes resulting from different curve patterns were analyzed and genotyped (Figure 1). Samples of the three observed curves were sequenced (ABI 3500XL Sequencer^®^, Applied Biosystems, Foster City, CA, USA) to confirm the genotypes indicated by HRM.

Quality control for the *APOE *polymorphisms (rs7412 and rs429358) was assessed by randomly selecting 96 samples to be re-genotyped using a high resolution melting method (HRM analysis, Rotor Gene 6000^®^, Qiagen, Courtaboeuf, France) by two independent technicians and these results were 100% concordant.

### Statistical Analysis

To evaluate the effect of *APOE *genotypes on cardiovascular risk factors, subjects were categorized into three groups: ε2 carriers (ε2/ε2 + ε2/ε3 genotypes), ε3 carriers (ε3/ε3 genotype) and ε4 carriers (ε4/ε4 + ε3/ε4 genotypes)[34]. In each model, the homozygous ε3/ε3 genotypes formed the reference group. Thirty-two individuals (2.3%) with ε2/ε4 genotype were excluded from the analysis because of the putative opposite effects of these two alleles on LDL-C levels [35].

Differences in the baseline cardiovascular risk characteristics across the *APOE *groups were tested by One-Way ANOVA for continuous variables, and χ^2 ^test for categorical parameters. Table 1 shows the comparison of data among all allelic groups. All variables were adjusted for ethnicity, age and gender, except the PWV was adjusted for age, SBP and ethnicity.

**Table 1:** Baseline characteristics of participants encoding ε2, ε3 and ε4 alleles of the APOE polymorphism.

Subject Characteristics n, (%)	ε2 180 (12.3%)	ε3 893 (61.1%)	ε4 388 (26.6%)	*p *value
**Gender, male**	76 (42.2%)	433 (48.5%)	169 (43.6%)	0.13
**Ethnicity**				
**African descendent**	19 (15.6%)	63 (51.6%)	40 (32.8%)	
**Caucasian descendent**	50 (9.1%)	367 (67.0%)	131 (23.9%)	0.002
**Mulatto**	111 (14.1%)	463 (58.5%)	217 (27.4%)	
**Smoking status, smokers (%)**	43 (23.9%)	211 (23.6%)	101 (26.0%)	0.66
**Diabetes (%)**	13 (7.2%)	70 (7.8%)	34 (8.7%)	0.81
**Age, years**	43.8 ± 10.6	45.1 ± 10.9	44.5 ± 10.8	0.28
**BMI, kg/m2**	25.8 ± 4.7	26.3 ± 4.9	26.4 ± 4.9	0.27
**SBP, mmHg**	125.9 ± 19.7	128.1 ± 21.5	129.4 ± 22.6	0.15
**DBP, mmHg**	83.3 ± 13.9	84.5 ± 14.1	85.1 ± 14.1	0.34
**PP, mmHg**	42.6 ± 11.4	43.7 ± 13.7	44.3 ± 14.3	0.34
**MBP, mmHg**	97.5 ± 15.2	99.0 ± 15.8	99.8 ± 16.1	0.21
**PWV, m/s**	10.0 ± 2.1	9.8 ± 2.2	9.8 ± 2.1	0.66
**Triglycerides, mg/dL**	133.7 ± 99.1	133.9 ± 124.4	145.9 ± 148.6	0.28
**Total cholesterol, mg/dL**	201.4 ± 50.6	215.2 ± 48.3*	218.6 ± 44.6†	< 0.001
**HDL - C, mg/dL**	48.8 ± 14.6	45.5 ± 12.7*	43.2 ± 10.1†‡	< 0.001
**LDL - C, mg/dL**	125.3 ± 35.8	143.6 ± 38.9*	147.9 ± 39.6†	< 0.001
**VLDL - C, mg/dL**	25.3 ± 17.2	25.5 ± 22.9	26.2 ± 15.5	0.81
**Total cholesterol/HDL - C ratio**	4.3 ± 1.4	5.0 ± 1.5*	5.3 ± 1.5†	< 0.001
**LDL - C/HDL - C ratio**	2.8 ± 1.0	3.4 ± 1.2*	3.6 ± 1.3†‡	< 0.001
**Glucose, mg/dL**	103.9 ± 30.1	105.8 ± 33.0	104.4 ± 31.0	0.64
**Creatinine, mg/dL**	0.99 ± 0.19	0.97 ± 0.20	0.96 ± 0.20	0.16
**Uric acid, mg/dL**	4.7 ± 1.4	4.8 ± 1.5	5.0 ± 1.6†	0.04

BMI, body mass index; SBP, systolic blood pressure; DBP, diastolic blood pressure; PP, pressure pulse; MBP, mean blood pressure; PWV, pulse wave velocity; HDL - C, high-density lipoprotein cholesterol; LDL - C, low-density lipoprotein cholesterol; VLDL - C, very-low-density lipoprotein cholesterol.

ε2 allele = ε2/ε2 + ε2/ε3 genotypes; ε3 allele = ε3/ε3 genotype; ε4 allele = ε3/ε4 + ε4/ε4 genotypes

* ε2 vs ε3, p < 0.05; † ε2 vs ε4, p < 0.05; ‡ ε3 vs ε4, p < 0.05.

All values were adjusted for ethnicity, age and gender except the PWV was adjusted for age, SBP and ethnicity.

Logistic regression analysis was carried out to estimate the odds ratio (OR), with 95% confidence intervals (CI), in order to assess genetic risk factors for common cardiovascular risk. The risk analysis was performed comparing all three groups together. In the Table 2, we present risk estimative for E2 and E4 allele carriers against all other allele groups.

**Table 2:** Analysis of the cardiovascular risk factors associated with APOE polymorphism

	**APOE ALLELES**					
	
	**ε2**			**ε4**		
		
**VARIABLES**	**OR**	**CI 95%**	***p *value**	**OR**	**CI 95%**	***p *value**
**Total cholesterol**	0.44	0.319 - 0.618	< 0.001	1.571	1.218 - 2.026	0.001
**HDL - C**	0.584	0.400 - 0.854	0.005	1.271	0.982 - 1.644	0.07
**LDL - C**	0.401	0.262 - 0.614	< 0.001	1.738	1.343 - 2.250	< 0.001
**Uric Acid**	0.500	0.237 - 1.056	0.07	1.748	1.170 - 2.611	0.006
**SBP**	0.786	0.526 -1.173	0.13	1.140	0.858 - 1.515	0.37
**DBP**	1.096	0.776 - 1.549	0.60	0.968	0.745 - 1.257	0.81
**BMI**	0.717	0.466 - 1.104	0.13	1.358	1.019 - 1.811	0.04

Adjusted values for ethnicity, gender and age.

Quantitative variables were expressed as the mean ± standard deviation, while qualitative variables were expressed as percentage. Hardy-Weinberg equilibrium for the distribution of the genotype groups was estimated using the Haploview software. All statistical analyses were carried out using SPSS software (v. 16.0), with the level of significance set at p < 0.05.

## Results

The frequency of *APOE *genotypes among 1461 participants were: ε2/ε2 - 0.4%, ε2/ε3 - 9.7%, ε2/ε4 - 2.3%, ε3/ε3 - 61.0%, ε3/ε4 - 24.4% and ε4/ε4 - 2.2%. The allele frequencies were: ε2 - 10.1%, ε3 - 61.0% and ε4 - 26.6%. The genotype distributions for *APOE *polymorphisms (rs7412 and rs429358) were in Hardy-Weinberg equilibrium (p > 0.05). The ethnicity proportion was different among the *APOE *allele groups (p = 0.002), where the number of Caucasians is higher than Afro-descendent in the ε3 allele group (67.0% vs. 51.6%, respectively). Comparative analysis failed to indicate a significant difference among *APOE *groups and age (p = 0.28), gender (p = 0.13), diabetes (p = 0.81) and smoking status (p = 0.66) (Table 1).

### Association Between Hemodynamic Phenotypes and APOE Polymorphism

Hemodynamic phenotypes of SBP (p = 0.15), DBP (p = 0.34), MBP (p = 0.21), PP (p = 0.34) and PWV (p = 0.66) showed no association with *APOE *allele groups (Table 1). Similarly, risk analysis performed by multiple logistic regression failed to detect significant genetic risk for high SBP (ε2 carriers: OR = 0.786, 95% CI = 0.526-1.173, ε4 carriers: OR = 1.140, 95% CI = 0.858-1.515) and high DBP (ε2 carriers: OR = 1.096, 95% CI = 0.776-1.549) and ε4 carriers: OR = 0.968, 95% CI = 0.745-1.257) (Table 2).

### Association Between Biochemical and Metabolic Phenotypes and APOE Polymorphism

Biochemical measurement of TC, LDL-C, HDL-C, TC/HDL ratio and LDL-C/HDL and uric acid were associated with the *APOE *polymorphism, even after adjustment for ethnicity, age and gender. Values of TC (p < 0.001), LDL-C (p < 0.001), TC/HDL ratio (p < 0.001) and LDL/HDL-C ratio were higher in ε4 and ε3 when compared to ε2 allele carriers. However, for TC, LDL-C and TC/HDL-C no difference was observed between ε2 and ε3 alleles (p > 0.05). The HDL-C values (p < 0.001) were lower in ε4 and ε3 alleles when compared with ε2 allele carriers. In addition, ε3 carriers showed higher HDL-C values when compared with ε4 allele carriers. The uric acid values (p = 0.04) were lower in ε2 allele when compared with ε4 allele carriers. Other variables, such as TG (p = 0.28), VLDL-C (p = 0.81), glucose (p = 0.64), creatinine (p = 0.16) and BMI (p = 0.27) failed to show association with *APOE *groups (Table 1).

The ε2 allele confers protection for high TC (OR = 0.444, 95% CI = 0.319-0.618), low HDL-C (OR = 0.584, 95% CI = 0.400-0.854), high LDL-C (OR = 0.401, 95% CI = 0.262-0.614), while ε4 allele confers risk for elevated levels of TC (OR = 1.571, 95% CI = 1.218-2.026), LDL-C (OR = 1.738, 95% CI = 1.343-2.250), uric acid (OR = 1.748, 95% CI = 1.170-2.611) and BMI (OR = 1.358, 95% CI = 1.019-1.811) (Table 2).

## Discussion

The main finding of the present study was that *APOE *genetic variability is associated with cardiovascular risk factors in a Brazilian urban population. Increased LDL-C, TC, TC/HDL-C ratio, LDL-C/HDL-C ratio and decreased HDL-C values were observed in individuals harboring the ε4 allele. However, this genetic variant was not associated with the arterial stiffness phenotype.

Corroborating our results, Medina-Urrutia et al[13] demonstrated associations between the ε4 allele of the *APOE *polymorphism and higher concentrations of TC, LDL-C and lower HDL-C levels when compared with the ε2 allele carriers in Mexican adolescents. Similarly, Shu Liang et al[36] studying 168 healthy Chinese individuals showed associations between the ε4 allele with increased TC and LDL-C values. However, HDL-C was not associated with the genetic variant studied. In the Brazilian population, de-Andrade et al[37] studying individuals of both genders described associations between the ε4 allele and higher TG and total and non-HDL levels only in women. Additionally, Mendes-Lana et al[38] showed that ε4 allele carriers present increased risk for dyslipidemia when compared to ε2 allele carriers. However, De França et al[39] failed to demonstrate an association between the ε4 allele with increased TC and LDL-C values in healthy children, suggesting that at least part of the described effect is age-dependent. In fact, although a large number of studies show associations between *APOE *polymorphisms with cardiovascular risk and lipid profile phenotypes in different populations [40,41], some studies failed to demonstrate this association [42,43].

Some functional studies may help in explaining our findings. Miettinen et al[44] found a significant higher cholesterol absorption in subjects with ε3 and ε4 alleles compared with those carrying the ε2 allele after a normal diet. In addition, Weintraub et al[45] showed that the slower hepatic clearance of dietary fat in ε2/3 subjects could result in up-regulation of LDL receptors and a subsequent decrease in plasma LDL-C levels.

Arterial stiffness and hypertension are the most important risk factors for cardiovascular diseases [2,46]. Several studies have demonstrated association of these with the metabolic profile [47,48]. The present study failed to demonstrate an association between *APOE *polymorphisms with blood pressure phenotypes and arterial stiffness. Corroborating some results of this study, Fuzikawa et al[49]studied 1406 Brazilian elderly individuals and found no association between *APOE *genotype and hypertension. Similarly, Carmo-Martins et al[50] studied 672 Portuguese subjects and failed to demonstrated an association with blood pressure.

Focusing on the arterial stiffness, studies[51] have showed that the unfavorable lipid profile is associated with lower arterial complacence due to reduced NO bioavailability induced by dyslipidemia. Thus, we expected ε4 allele carriers of the *APOE *polymorphism would have a higher arterial stiffness when compared to ε2 allele carriers. However, this was not evident in this study, perhaps due to the little difference in mean lipid fractions between the groups as well as the relative young age of studied individuals. Studies involving the *APOE *polymorphism with arterial stiffness phenotype remain scarce in the literature. Thereby, further studies involving the *APOE *polymorphism with arterial stiffness phenotype are needed to clarify these issues.

Classically, obesity and hyperuricemia have shown to have important roles in the development of cardiometabolic disease [3,52,53]. This study showed that ε4 allele is associated with higher uric acid levels when compared to ε2 allele carriers. In addition, the presence of the ε4 allele offered increased risk for obesity and hyperuricemia. Unlike our findings, Liberopoulos et al[54] studying healthy individuals showed that the ε4 allele was associated with lower uric acid levels when compared to allele ε2. Similar to our study, ε2 allele was associated with lower serum levels of TC. Among the mechanisms that may explain the higher prevalence of hyperuricemia in individuals carrying the ε4 allele of *APOE *polymorphism in our study could be the hyperinsulinemia associated with an unfavorable lipid profile. Several studies[55] have confirmed that under-excretion of uric acid into the urine caused by the effect of insulin on the urinary tubular tract has been demonstrated with physiological hyperinsulinemia acutely reducing urinary uric acid which could lead to a higher concentration of this protein in the bloodstream. Due to the controversial results and few existing data, studies of the association between *APOE *polymorphism and uric acid need to be further conducted. Similarly, studies associating obesity phenotypes with *APOE *polymorphism have shown controversial results. Corroborating our results, Carmo-Martins et al.[50] showed that obese subjects (BMI ≥ 30 Kg/m^2^) are more prevalent in the ε4 allele group than in the ε2 allele group. In another study, Kolovou et al[56] showed in coronary heart disease patients that the prevalence of obese individuals was higher in the ε4 allele than in the ε2 allele group. Nevertheless, other studies failed to show this association [57]. The relationship between dyslipidemia and obesity is well established in the literature. However, the mechanism by which apolipoprotein E would influence obesity is not clear. Karagiannides et al[58] showed through an experimental study that rat ApoE3^knock in ^after a high-fat diet gained more weight than animals that did not express the APOE protein and animals that expressed isoform APOE2. Considering that *in vitro *receptor binding studies established that lipid-bound ApoE3 and ApoE4 have a similar affinity for LDLr, whereas lipid-bound ApoE2 has a much lower affinity[59], we hypothesize that this higher affinity of APOE4 isoform could result in increased lipid deposition in the adipose tissue.

Our study has potential limitations. First, it should be noted that the relative young age of the studied population may preclude the identification of the association between the studied genotype and measures of arterial stiffness. Second, we did not evaluate the use of cholesterol-lowering drugs, which could confound the observed associations. Finally, if we have measured insulin and urinary uric acid levels in this population, we could shed light into the proposed mechanism for the association of *APOE *polymorphism and serum uric acid.

In conclusion, the present study confirms the close association between *APOE *polymorphism and the lipid profile in individuals from the general population, but failed to show this genetic risk factor as an important modulator of arterial distensibility in this same sample.

## Competing interests

The authors declare that they have no competing interests.

## Authors' contributions

***ROA ***and ***SRSF ***participated in the design of the study, performed the statistical analysis and drafted the manuscript. ***PCJLS ***and ***NEF ***contributed to acquisition of data and its interpretation. ***RSC**, **JGM ***and ***JEK ***contributed to conception and design of the study. ***ACP ***conceived of the study, participated in its design, coordination and helped to draft the manuscript. All authors read and approved the manuscript.
